# Long-term psychological intervention for parents of children with prolonged disorders of consciousness: a pilot study

**DOI:** 10.3389/fpsyg.2023.1212014

**Published:** 2023-11-30

**Authors:** Gang Xu, Fuchun Hao, Weiwei Zhao, Peng Zhao, Jiwen Qiu

**Affiliations:** ^1^Rehabilitation Branch, Tianjin Children’s Hospital/Children’s Hospital, Tianjin University, Tianjin, China; ^2^Tianjin Key Laboratory of Birth Defects for Prevention and Treatment, Tianjin Children’s Hospital/Children’s Hospital, Tianjin University, Tianjin, China; ^3^Tianjin Medical College, Tianjin, China; ^4^Tianjin Beichen Experimental Middle School, Tianjin, China; ^5^Research Center of Experimental Acupuncture Science, Tianjin University of Traditional Chinese Medicine, Tianjin, China; ^6^School of Medical Technology, Tianjin University of Traditional Chinese Medicine, Tianjin, China

**Keywords:** psychological intervention, prolonged disorders of consciousness, parent, family, children

## Abstract

**Background:**

Children with prolonged disorders of consciousness experience severe intellectual and behavioral disabilities that will last for decades or even a lifetime. Parents generally experience severe anxiety, stress, sadness, or family conflicts, which can lead to abnormal parenting behavior and can, in turn, adversely affect the cognitive, emotional, and behavioral well-being of the children. This causes a serious burden on children, families, and society. Psychological interventions targeting parents using online conversations provide an opportunity to improve the overall well-being of the parents, their children, and the family as a whole.

**Methods:**

A total of 13 patients completed the protocol. Six were girls (46.2%), the mean age was 4.5 ± 3.0 years, and the length of time before emergent from minimally consciousness state was 244 ± 235 days. A staff member with psychological counseling qualifications implemented all psychological interventions. Regular online psychological interventions were performed annually before and after discharge. Evaluation data were collected before discharge and at 1 and 3–5 years post-discharge.

**Results:**

With the extension of intervention time, the Strengths and Difficulties Questionnaire, the Depression Anxiety and Stress Scale-21, and the Parenting Sense of Competence Scale scores showed significant improvement (*p* < 0.05), while the Revised Scale for Caregiving Self-Efficacy scores did not. With the extension of intervention time, the Strengths and Difficulties Questionnaire (Total Difficulties scores, TD) scores showed significant improvement (*p* < 0.05), while the scores did not after 1 year compared with before intervention. The Index of Child Care Environment evaluation scores declined significantly (*p* < 0.05).

**Conclusion:**

Psychological interventions aimed at the parents of children with prolonged disorders of consciousness performed at least once per year resulted in significant improvements in negative parental emotions, parental self-efficacy, and emotional and behavioral problems in their children. However, the childcare environment continued to decline.

## Introduction

Prolonged disorders of consciousness (PDoC) refer to consciousness-related disorders characterized by a loss of consciousness for longer than 28 days (Giacino et al., 2018; Kondziella et al., 2020). However, there is little pediatric-specific evidence and associated clinical recommendations regarding the natural history and prognosis of PDoC in children and adolescents, and no established treatment options exist for pediatric patient populations (Giacino et al., 2018). Children affected by PDoC often face severe behavioral and intellectual disabilities. This can make it very difficult for parents raising children impacted by PDoC to appropriately balance caring for their children with other priorities, leading to severe stress, anxiety, sadness, or familial conflict that can contribute to abnormal parenting behavior. These problems can, in turn, further exacerbate the emotional, behavioral, and cognitive problems in the children, ultimately imposing a significant burden on the children, their families, and society. Caregivers of patients in a PDoC state often experience a low quality of life and significant distress. Therefore, it is essential to establish appropriate support systems to assist these caregivers adequately (Gosseries et al., 2023). Despite the potentially serious long-term consequences, children with PDoC currently do not receive systematic and comprehensive rehabilitation treatment, posing a risk of poor health outcomes and reduced quality of life.

Psychological interventions for children with brain injury involve mainly four stages, namely, interventions addressing disorders of consciousness, cognitive state, acute cognitive intervention, and follow-up care (Xu et al., 2022; Watson et al., 2023). The first three stages are mainly carried out in hospitals, while the last stage occurs in families, schools, and communities. Each stage of rehabilitation involves different cognitive, emotional, and behavioral challenges. Therefore, a dynamic evaluation of psychological changes and appropriate adjustment of intervention methods is necessary. In an inpatient environment, communication, and coordination between children, parents, and counselors can be carried out quickly and effectively. However, this becomes considerably more difficult in a family environment. Moreover, the family environment represents the long-term living environment for both children and parents. Fortunately, the children with acquired brain injuries that have the best outcomes are those who are enthusiastic and willing to communicate, and also have parents with lower levels of passivity, indulgence, and authoritarianism (Yeates et al., 2010; Micklewright et al., 2012). Training in parental skills may be effective in improving parent, family, and couple outcomes in families of children with acquired brain injuries (Brown et al., 2015). Meanwhile, long-term longitudinal research provides the most effective foundation for efforts to understand the complex adaptation process undergone by children and their families after a child sustains a severe head injury (Xu et al., 2020). Petranovich and colleagues found that psychological intervention for six months promoted a long-term reduction in psychological pain but had little effect on depressive symptoms and the self-efficacy of caregivers (Petranovich et al., 2015). Remote online psychological counseling was welcomed as a psychological intervention tool during the COVID-19 pandemic (Galvin et al., 2023). Moeller et al. found psychotherapy by video conferencing to be effective, especially if patients and counselors have already established a good treatment relationship before online interaction (Moeller et al., 2022). Counselor-assisted problem-solving provides a structured yet flexible approach to caregiver self-efficacy (Wade et al., 2014) and addresses the executive dysfunction of children (Kurowski et al., 2013) that often accompanies traumatic brain injury. Therefore, more attention should be paid to psychological intervention in the family environment, especially for interventions lasting for more than six months and targeting parents online. As such, psychological interventions targeting parents provide an opportunity to improve the overall well-being of the parents, their children, and the family as a whole. However, no similar psychological interventional methods have been reported in the context of children with PDoC.

We hypothesized that psychological intervention targeting parents can improve both the well-being of children and parents for three to 5 years. We thus provided parents with active, long-term psychological counseling services following the hospitalization and discharge of children with PDoC to monitor both psychological changes in the parents and associated changes in the family environment. In this case series, we report the results of the use of psychological interventions for the parents of 16 children with PDoC over a three-to-five-year follow-up interval to provide a reference for the optimal long-term management of PDoC in children.

## Methods

### Participants and procedure

Recruitment for this study was performed from July 2016 to January 2020 through the Rehabilitation Medicine Department of Tianjin Children’s Hospital (Tianjin Children’s Rehabilitation Center) in China. The inclusion criteria were: (a) children who had been diagnosed with PDoC and had emerged from a minimally conscious state prior to discharge as assessed by the Coma Recovery Scale for Pediatrics (CRS-P) (Slomine et al., 2019) and the Glasgow Coma Scale (GCS) (Mehta and Chinthapalli, 2019); (b) the parents or caregivers of the children had agreed to participate and cooperate with long-term follow-up. The exclusion criteria included: (a) parents or caregivers who had been diagnosed with intellectual disability, depression, or anxiety; (b) children who had been diagnosed with intellectual disability before being diagnosed with PDoC; (c) children with progressive deterioration of the consciousness disorder. In total, 16 families were recruited, of which two were lost to follow-up as their phone numbers had changed and one did not agree to continue participating. The demographics and clinical characteristics of the participants are shown in Tables 1, 2. The study was approved by the Ethics Committee of the Tianjin Children’s Hospital. Before participating, the participants’ legal guardians provided signed informed consent.

**Table 1:** Characteristics of the children.

Sex	Age of onset (yr)	Diagnosis	First GCS (E-V-M)	Duration of coma (d)	From onset to MCS (d)	From onset to EMCS (d)	Epilepsy	Previous development	3 ~ 5 years after discharge	
									Development Quotient OR intelligence quotient	Academic situation (now)
Boy	1	Intracranial hemorrhage (trauma)	1-1-1	38	87	136	NO	Age appropriate	GDDS: adaption46; gross motor20;fine motor 58; language 54; social 49	Unable to attend school
Girl	3	Intracranial hemorrhage (trauma)	1-1-2	24	493	803	Yes	Age appropriate	GDDS: adaption 15; gross motor 14; fine motor 15; language 13; social 14	Unable to attend school
Girl	7	Intracranial hemorrhage (vascular malformation)	1-1-1	8	98	131	No	Age appropriate	65 (WISC-CR): IQV 74, QP 61	Fourth grade of primary school (12-year old)
Girl	3	Acute necrotizing encephalitis	1-1-2	8	30	63	No	Age appropriate	GDDS: adaption 51; gross motor 53; fine motor 49; language 48; social 49	Unable to attend school
Girl	5	Encephalomyelitis	1-1-3	5	90	165	Yes	Age appropriate	GDDS: adaption33; gross motor 33; fine motor 22; language 40; social 26	Unable to attend school
Girl	6	Encephalomyelitis	1-1-2	12	66	94	No	Age appropriate	60 (WISC-CR): IQV 68, IQP 60	Third grade of primary school (11-year old)
Boy	11	Purulent meningitis	1-2-4	36	182	423	Yes	Age appropriate	GDDS: adaption31; gross motor 45; fine motor 20; language 29; social 33	Unable to attend school
Boy	6	Intracranial hemorrhage (trauma)	2-2-4	1	29	29	No	Age appropriate	90 (WISC-CR): IQV 89, IQP 92	Fourth grade of primary school (10-year old)
Boy	2	Poisoning (AB adhesive, epoxy resin)	1-1-1	3	320	566	Yes	Age appropriate	GDDS: adaption35; gross motor 50; fine motor 23; language 25; social 30	Unable to attend school
Girl	5	Intracranial hemorrhage (trauma)	2-1-3	12	31	32	No	Age appropriate	93 (WISC-CR): IQV 95, IQP 92	Third grade of primary school (9-year old)
Boy	1	Hypoxic ischemic encephalopathy (postoperative tetralogy of fallot)	1-1-2	8	125	458	Yes	Age appropriate	GDDS: adaption45; gross motor 54; fine motor 50; language 37; social 40	Unable to attend school
Boy	8	Intracranial hemorrhage (trauma)	1-1-1	9	30	31	No	Age appropriate	88 (WISC-CR): IQV 85, IQP 94	Five grade of primary school (11-year old)
Boy	1	Intracranial hemorrhage (trauma)	1-1-2	6	114	239	Yes	Age appropriate	GDDS: adaption22; gross motor 34; fine motor 21; language 29; social 36	Unable to attend school

GCS: Glasgow coma scale (Eye opening-Verbal response-Motor response); GDDS: Gesell Development Diagnosis Scale (Chinese version); WISC-CR: Wechsler Intelligence Scale for Children-Chinese Revised, verbal intelligence quotient (IQV) and performance intelligence quotient (IQP).

**Table 2:** Family characteristics.

Parent relationship to child	Relationship status	Participating parent’s education	Participating parent’s employment	Family annual income (AUD)	Parent receiving assistance
Mother	Married	Trade/college	Home-duties/ unemployed	50,000 to 120,000	Psychological consultant
Aunt	Widowed	<High school diploma	Home-duties/ unemployed	50,000 to 120,000	Psychological consultant
Mother	Married	Trade/college	Home-duties/ unemployed	> 120,000	Psychological consultant
Mother	Married	Trade/college	Full-time	50,000 to 120,000	Psychological consultant
Mother	Married	College degree	Part-time	50,000 to 120,000	Psychological consultant
Mother	Married	College degree	Part-time	> 120,000	Psychological consultant
Mother	Married	<High school diploma	Part-time	<50,000	Psychological consultant
Grandfather	Widower	<High school diploma	Part-time	<50,000	Psychological consultant
Mother	Married	Trade/college	Part-time	50,000 to 120,000	Psychological consultant
Mother	Married	College degree	Full-time	> 120,000	Psychological consultant
Mother	Divorce	<High school diploma	Part-time	<50,000	Psychological consultant
Mother	Divorce	<High school diploma	Part-time	50,000 to 120,000	Psychological consultant
Mother	Married	College degree	Home-duties/ unemployed	>120,000	Psychological consultant

Five online questionnaires that were designed and published on the Chinese Star Survey website were used.^1^ Evaluation data were collected before discharge and at 1 and 3–5 years post-discharge. The psychological counselors adjusted the psychological intervention measures based on the results of the questionnaires. To ensure the privacy of the participants and the credibility of the information obtained, the investigators undertook, at the time of recruitment, not to disclose personal information to any person or organization. The psychological counselor provided one-on-one online counseling to these family members. Regular online psychological interventions were performed once every year before and after discharge, and families were provided with the option for irregular online appointment-based psychological counseling if they encountered any psychological problems or otherwise felt the need for counseling. Each Health Psychological Intervention lasted for 1 hour using Tencent Meeting (Tencent Meeting is an audio and video conferencing software under Tencent Cloud, https://voovmeeting.com/).

The counselors were trained and qualified psychology professionals who were experienced in reducing abnormal parenting behaviors by providing Health Psychological Interventions (when a normal person experiences emotional problems such as anxiety, tension, fear, and depression caused by various stimuli, or problematic behaviors caused by setbacks that affect the functioning of society, health psychological counseling is provided). Our team’s two psychological counselors, with discussion with departmental leaders, designed an online psychological intervention plan. The components of the online psychological intervention included: ① Mindfulness training, where participants were provided with audio instructions for body scans and sitting meditation to guide them through the exercises; ② Psychoeducation, where the psychological counselors assisted parents in enhancing their self-efficacy and alleviation of psychological stress by releasing Psychological Popular Knowledge on WeChat; ③ Family Education Guidance, which assists families in establishing a good family environment in terms of marital and parent–child relationships, and thus helps families to be happy.

### Measures

1. The Strengths and Difficulties Questionnaire (SDQ): The Strengths and Difficulties Questionnaire is a 25-item instrument designed to assess emotional and behavioral problems. The Cronbach’s α was 0.784. This questionnaire generates scores over five areas of children’s psychological adjustment: hyperactivity-inattention, emotional symptoms, prosocial behavior, conduct problems, and peer problems (Dickey and Blumberg, 2004; Kou et al., 2005).
2. The Revised Scale for Caregiving Self-Efficacy (RSCSE): The Revised Scale for Caregiving Self-Efficacy is designed to identify caregivers who have experienced extensive psychosocial distress. The Cronbach’s α coefficients were all greater than 0.80. The project is arranged in the order of the average difficulty level in each subscale, from easiest to hardest (Cheng et al., 2013; Steffen et al., 2019). This measurement strategy offers a simple and effective way to assess caregiver self-efficacy.
3. The Depression Anxiety and Stress Scale-21 (DASS-21): The Depression Anxiety and Stress Scale-21 includes the subscales of depression (*α* = 0.97), anxiety (*α* = 0.94), and pressure (*α* = 0.95). The higher the score, the more severe the symptoms (Osman et al., 2012; Lu et al., 2018).
4. The Parenting Sense of Competence Scale (PSOC): The Parenting Competence Scale has the potential to be used as a clinical and research instrument for measuring maternal role competence and satisfaction in the Chinese population. The scale consists of 17 items and has 2 subscales, namely, skills/knowledge and values/comfort (Ngai et al., 2007). The total score is 17–102, and the higher the score, the higher the overall ability of parenting. The Cronbach’s α coefficient of the whole scale, the efficacy subscale, and the satisfaction subscale in the Chinese version were 0.872, 0.802, and 0.874, respectively (Li et al., 2021).
5. The Index of Child Care Environment (ICCE): The ICCE measures the childcare environment through 13 questions with four subscales. The correlation coefficients for “total score,” “human stimulation,” “social stimulation,” “avoidance of restriction,” and “social support” were 0.76, 0.78, 0.82, 0.82, and 1.00, respectively (Anme et al., 2013). The higher the score, the better the childcare environment (Gao et al., 2021). Scores are divided into four groups: the worst family parenting environment group (≤ 10 points), the lower middle group (11 points), the upper middle group (12 points), and the best group (13 points).
6. The Gesell Development Diagnosis Scale (GDDS): The Gesell Development Diagnosis Scale encompasses five domains: adaptability, gross motor, fine motor, language, and social personality. The internal consistency coefficients range from 0.80 to 0.93 (Hutt and Hutt, 1970). GDDS is not only used to evaluate the neuropsychological development of children aged 0–6 years but is also one of the standardized methods for evaluating intellectual disabilities in children aged 0–6 years. The Chinese version of the GDS has been widely used in previous research with good internal consistency, reliability, and validity (Liu et al., 2016; Chen et al., 2020). The DQ scores are divided into mild (55 ≤ DQ < 75), moderate (40 ≤ DQ < 55), severe (25 ≤ DQ < 40), and suspected (75 ≤ DQ < 84) neurodevelopmental disability.
7. The Wechsler Intelligence Scale for Children–Chinese Revised (WISC-R): This is the Chinese version of the Webster Intelligence Scale for Children, which has been proven to be suitable for evaluating the intellectual development of Chinese school-age children. Reliability coefficients were between 0.68 and 0.98 (Li et al., 1990; Zhou et al., 2020). This scale is composed of a verbal intelligence quotient (IQV) and a performance intelligence quotient (IQP).

### Statistical analyzes

1. Descriptive statistics and changes over time in SDQ, RSCSE, DASS-21, PSOC, and ICCE were reported using means, standard deviations, and percentage delta. Percentage delta = [(y−x)/x] × 100%. T1 = [(1 year after discharge−the day of discharge)/the day of discharge]×100%; T2 = [(3~5years after discharge−the day of discharge)/the day of discharge]×100%.
2. SPSS v23.0 (version 23.00; IBM Corp, Armonk, NY, United States) was used for the statistical analysis. The Shapiro–Wilk test was applied to determine whether the random sample came from a population with a normal distribution. A normal distribution of variables was tested using paired two-tailed t-tests; the Wilcoxon-signed rank test was applied for non-normally distributed variables. *p*-values <0.05 were considered statistically significant.

## Results

**Figure 1:**
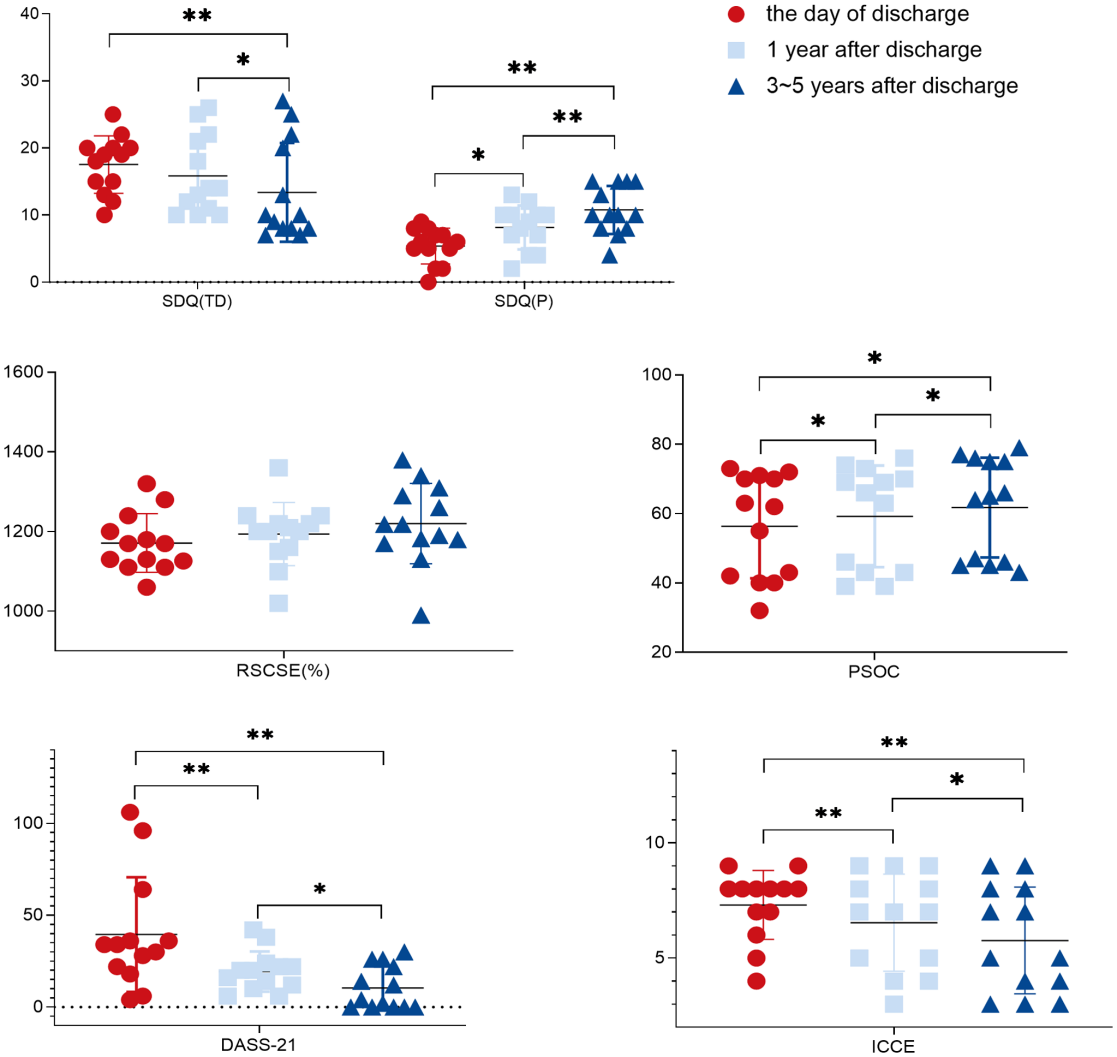
1. SDQ (TD), strengths and difficulties questionnaire (Total Difficulties scores); SDQ (P), strengths and difficulties questionnaire (Prosocial subscale score); RSCSE, Revised Scale for Caregiving Self-Efficacy; DASS-21, Depression Anxiety and Stress Scale-21; PSOC, Parenting Sense of Competence Scale; ICCE, Index of Child Care Environment. 2. *: *p* < 0.05; **: *p* < 0.01.

**Figure 2:**
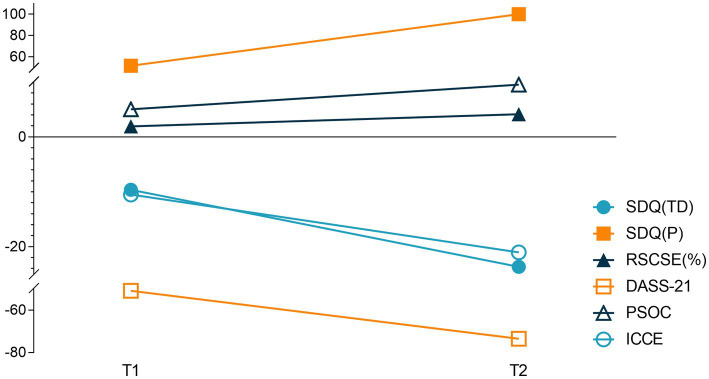
T1 = [(1 year after discharge−the day of discharge) / the day of discharge] × 100%; T2 = [(3 ~ 5 years after discharge−the day of discharge) / the day of discharge] × 100%.

All children included in this case series exhibited severe intellectual or cognitive disabilities at 3–5 years after emerging from a minimally conscious state. Just five of these children were enrolled in school. Moreover, 85% of the mothers of these children were either unable to work or were only able to work part-time (Tables 1, 2) with the prolongation of follow-up. Outcome measures at different points were performed on the day of discharge, after 1 year, and after 3–5 years (Figure 1; Table 3). Outcome measures for percentage delta were performed on T1 and T2 (Figure 2; Table 4).

**Table 3:** Outcome measures at different points [ $\bar{x}$ ± S / Median (Quartiles)].

Scale	The day of discharge		1 year after discharge		3 ~ 5 years after discharge		t/z, *p*		
							①	②	③
RSCSE (%)	1171.23	73.55	1193.77	79.53	1219.85	100.85	−1.6, 0.14	−2.09, 0.06	−1.63, 0.13
PSOC	56.38	15.00	59.23	14.65	61.77	14.42	−2.3, 0.04	−2.63, 0.02	−2.32, 0.04
SDQ (TD)	17.54	4.27	15.85	5.86	13.38	7.35	1.56, 0.14	2.37, 0.04	3.18, 0.008
SDQ (P)	6	(3.5, 7.5)	9	(5.5, 10)	10	(8, 15)	−2.88, 0.04	−3.05, 0.002	−2.68, 0.007
DASS-21	34	(25, 50)	20	(11, 23)	4	(0, 24)	−2.98, 0.003	−3.06, 0.002	−2.05, 0.04
ICCE	7.31	1.49	6.54	2.11	5.77	2.31	2.38, 0.04	3.33, 0.006	2.25, 0.04

1. ① “the day of discharge” and “1 year after discharge”; ② “the day of discharge” and “3 ~ 5 years after discharge”; ③ “1 year after discharge” and “3 ~ 5 years after discharge.”

**Table 4:** Outcome measures for percentage delta.

	T1	T2
SDQ (TD)	13.89	32.41
SDQ (P)	55.26	110.53
RSCSE (%)	3.02	5.44
DASS-21	57.14	74.29
PSOC	1.67	5.00
ICCE	13.64	27.27

With the extension of intervention time, the SDQ, DASS-21, and PSOC scores showed significant improvement (*p* < 0.05), while the RSCSE and SDQ (TD) did not, after 1 year compared with before intervention, as shown in Figure 1 and Table 3. An upward trend in the RSCSE evaluation scores (1,500% of the total score), consistent with a gradual increase in parental caregiving self-efficacy, was observed. The ratio of the average value to the total score was >78%, reflecting a high degree of parental self-efficacy. The ICCE evaluation scores were all <10 points consistent with a poor parenting environment, and all scores declined significantly indicative of a gradual decline in the parenting environment in the families of these children (*p* < 0.05).

## Discussion

The incidence rate of vegetative state/unresponsive syndrome is estimated to be between 0.2/100000 and 6.1/100000 of the population (van Erp et al., 2014). Therefore, the incidence rate of PDoC is extremely low. It is also extremely rare for children to emerge from this state. This group of easily ignored people urgently needs our attention in the face of unimaginable difficulties. This case series is the first longitudinal analysis of the long-term effects of psychological interventions for the parents of children affected by PDoC over a 3–5 year follow-up period. With the extension of intervention time, the SDQ, DASS-21, and PSOC scores showed significant improvement (*p* < 0.05), while the RSCSE and SDQ (TD) scores did not, after one year compared with before intervention. However, the ICCE evaluation scores showed a significant decrease, indicative of a gradual decline in the parenting environment of the families of these children (*p* < 0.05) over the 3–5 year follow-up period.

Regarding the psychological distress of caregivers for patients with PDoC, 67 and 79% of respondents, respectively, reported thoughts of anxiety and depression, emphasizing once again the high level of psychological distress in this population (Gosseries et al., 2023). Psychological interventions have been shown to improve various disorders, including stress, anxiety, and emotional disorders (Cermak et al., 2022; LaRovere et al., 2022), as well as self-efficacy in the parents of children with acquired brain injuries (Cardile et al., 2023), consistent with the present results. Beliefs regarding self-efficacy have also been demonstrated to impact the onset of response, cost of effort, and behavioral persistence in challenging situations (Steffen et al., 2019). Amelioration of parental psychological disorders and improvements in self-efficacy can establish a positive feedback cycle with improvements in the social strength of their children. This study included a 3–5 year follow-up period, suggesting that psychological interventions performed at least once per year can facilitate continuous improvements in psychological disorders of parents, thereby maintaining a high degree of parental self-efficacy. At the same time, the emotional and behavioral problems in children affected by PDoC were also significantly improved.

Hiltonba found that disease uncertainty is a dynamic process wherein cognitive uncertainty transitions to ambiguity, leading to corresponding emotional changes with time that can include negative or positive attitudes (Hilton, 1994). Children affected by PDoC will experience severe behavioral and intellectual disorders that may last for years or even a lifetime. The uncertainty regarding their future, together with a lack of training in familial and parental guidance may account for the observed lack of significant improvement in the caregiver self-efficacy in the parents of this case series and the significant decline in the parenting environment. Research focused on the long-term management of children who have suffered moderate-to-severe head injuries has not reported any substantial positive effects associated with overall familial function (Shen et al., 2023). Current interventional approaches primarily focus on training cognitive, emotional, and behavioral coping by family members (Narad et al., 2017), but neglect interactions among family members (Moscato et al., 2021). The Family Process Model Theory posits that flexibility and adaptive interactions among seven different dimensions are required for appropriate familial function, including the dimensions of communication, involvement, behavior, task completion, emotional expression, shared values, and agreed-upon rules (Skinner et al., 2010). Improving familial parenting-focused knowledge may contribute to greater improvements in the sense of competence felt by parents, enhancing the family parenting environment as a whole. For instance, benefits may be derived from improvements in familial cohesion (Perrin et al., 2013), leading to better communication among the members of a family (Moscato et al., 2021).

## Study limitations

The present study has some important limitations that need to be considered. First, it analyzed only 13 patients with PDoC, which cannot exhaustively represent this clinical population. Second, demographic characteristics were not analyzed as potential risk factors affecting outcomes, and third, since there was no control group, we cannot determine whether the observed improvement in symptoms was the result of the intervention. However, we do not know which one of three aspects of the intervention was the most weighting on the outcome. Future studies should address these limitations by providing data from a larger sample of patients. Research should consider sociodemographic characteristics and other psychosocial conditions as potential factors influencing changes during the treatment.

## Conclusion

Psychological interventions aimed at the parents of children with PDoC performed at least once per year were associated with improvements in negative parental emotions, parental self-efficacy, and emotional and behavioral problems in their children. However, the childcare environment continued to decline. This may suggest that it is necessary to increase the frequency of family- and parenting-focused training to contribute to better long-term management of families affected by PDoC and their associated familial outcomes.

## Data availability statement

The original contributions presented in the study are included in the article/supplementary material, further inquiries can be directed to the corresponding authors.

## Ethics statement

The studies involving humans were approved by the Ethics Committee of the Tianjin Children’s Hospital. The studies were conducted in accordance with the local legislation and institutional requirements. Written informed consent for participation in this study was provided by the participants’ legal guardians.

## Author contributions

GX contributed to conception and design and drafted manuscript. FH and WZ contributed to acquisition and interpretation and drafted manuscript. PZ and JQ critically revised manuscript. All authors contributed to the article and approved the submitted version.

## Footnotes

^1^ https://www.wjx.cn/
